# Structural insights into cardiolipin transfer from the Inner membrane to the outer membrane by PbgA in Gram-negative bacteria

**DOI:** 10.1038/srep30815

**Published:** 2016-08-04

**Authors:** Haohao Dong, Zhengyu Zhang, Xiaodi Tang, Shihai Huang, Huanyu Li, Bo Peng, Changjiang Dong

**Affiliations:** 1 Biomedical Research Centre, Norwich Medical School, University of East Anglia, Norwich Research Park, Norwich, NR4 7TJ, UK; 2 Kennedy Institute of Rheumatology, Nuffield Department of Orthopaedics, Rheumatology and musculoskeletal Sciences, University of Oxford, Oxford, OX3 7FY, UK; 3 State Key Laboratory of Subtropical Bioresource Conservation and Utilization, Guangxi University, Nanning, Guangxi, 530004, China

## Abstract

The outer membrane (OM) of Gram-negative bacteria is a unique asymmetric lipid bilayer in which the outer leaflet is composed of lipopolysaccharide (LPS) and the inner leaflet is formed by glycerophospholipid (GPL). The OM plays a fundamental role in protecting Gram-negative bacteria from harsh environments and toxic compounds. The transport and assembly pathways for phospholipids of bacterial OM are unknown. Cardiolipin (CL) plays an important role in OM biogenesis and pathogenesis, and the inner membrane (IM) protein PbgA, containing five transmembrane domains and a globular domain in periplasm has been recently identified as a CL transporter from the IM to the OM with an unknown mechanism. Here we present the first two crystal structures of soluble periplasmic globular domain of PbgA from *S. typhimurium* and *E. coli*, which revealed that the globular domains of PbgA resemble the structures of the arylsulfatase protein family and contains a novel core hydrophobic pocket that may be responsible for binding and transporting CLs. Our structural and functional studies shed an important light on the mechanism of CL transport in Gram-negative bacteria from the IM to the OM, which offers great potential for the development of novel antibiotics against multi-drug resistant bacterial infections.

Gram-negative bacteria contain two membranes, the inner membrane (IM) and the outer membrane (OM) with the solvent filled periplasm and the peptidoglycan^1^,^2^. The peptidoglycan, which is much thinner than that found in Gram-positive bacteria, is bound to the OM by lipoproteins, but it remains strong and elastic to protect the bacteria against harsh environmental conditions^2^,^3^. The OM is an asymmetric lipid bilayer in which the inner leaflet is composed of glycerophospholipids (GPL) while the outer leaflet consists primarily of the glycolipid lipopolysaccharide (LPS), which functions as a barrier protecting bacteria from antibiotics and other harmful hydrophobic chemicals^1^,^2^,^4^,^5^. The OM also contains OM proteins and lipoproteins. The pathways of transport and assembly of LPS, lipoprotein and OMP from the IM to the OM have been identified and studied extensively; seven proteins LptA-G form a trans-envelope complex and transport LPS from the IM to the OM^1^,^2^,^6^,^7^,^8^,^9^,^10^,^11^,^12^,^13^. Five proteins LolA-E are responsible for sorting and transporting lipoproteins from the IM to the OM^14^,^15^,^16^,^17^, while five proteins BamA-E form a OMP insertion machine, which inserts unfolded OMP into the OM^18^,^19^,^20^,^21^,^22^. The proteins involved in phospholipid transport and assembly in the OM, however remain unknown.

In contrast, the IM is a symmetrical bilayer and is composed of phospholipids that maintain the barriers’ permeability for hydrophilic molecules and support the functions of the IM proteins^3^. The GPL molecules include phosphatidylethanolamine (PE), phosphatidylglycerols (PG) and cardiolipin (CL) in an approximately 75:20:5 ratio, respectively, in most enterobacterial membranes. CL is also known as diphosphatidylglycerol because it appears to contain two overlapping PG units (Supplementary Fig. S1). CL is also located in the OM of most Gram-negative bacteria, where it is essential for bacterial pathogenesis and survival in different environments under the regulatory control of the PhoPQ two-component system^23^,^24^. However, CL is synthesized at the IM, and so must be transported by lipid transfer proteins from the IM to the OM^25^,^26^.

Interestingly, CL is also presented in the IM of mitochondria, where it consists of about 20% of the total lipids, and is required for the mitochondrial function^27^,^28^. In yeast, the UPS1 and Mdm35 complex has been identified to transport phosphatidic acid (PA) from the mitochondria OM to the IM, and the structure of the UPS1 and Mdm35 complex has been recently solved^29^,^30^. In Gram-negative bacteria the Mla pathway, containing six proteins MlaA-F, has been suggested to be responsible for phospholipids transport from the OM to the IM^31^. In addition, the trigalactosyldiacylglycerol (TGD) pathway has been reported to deliver phosphatidic acid from the OM to the IM of chloroplasts^32^. However, there is no report about how the phospholipids are transported and assembled from the IM to the OM.

During infection, *Salmonella typhimurium* and *Escherichia coli* PhoPQ two-component regulatory system coordinates OM remodeling by activating genes encoding proteins and enzymes that increase OM-lipid hydrophobicity and decrease OM-lipid negative charge on the bacterial surface; this prevents binding and insertion of cationic antimicrobial peptides (CAMP) that kill the microbe^23^. *S. typhimurium* PbgA is an inner membrane protein containing five transmembrane helices and a globular periplasmic domain, which binds CL to promote PhoPQ-regulated trafficking of CL from the IM to the OM^24^.

Here we present the crystal structures of the globular domain of PbgA from *S. typhimurium* and *E. coli*, as well as functional assays. The structures and functional assays revealed the molecular basis of how the globular domain transports CL from the IM to the OM. To our knowledge, this is the first protein structure reported involved in phospholipid transport from the IM to the OM in bacteria.

## Results

### Two structures of PbgA

**Figure 1:**
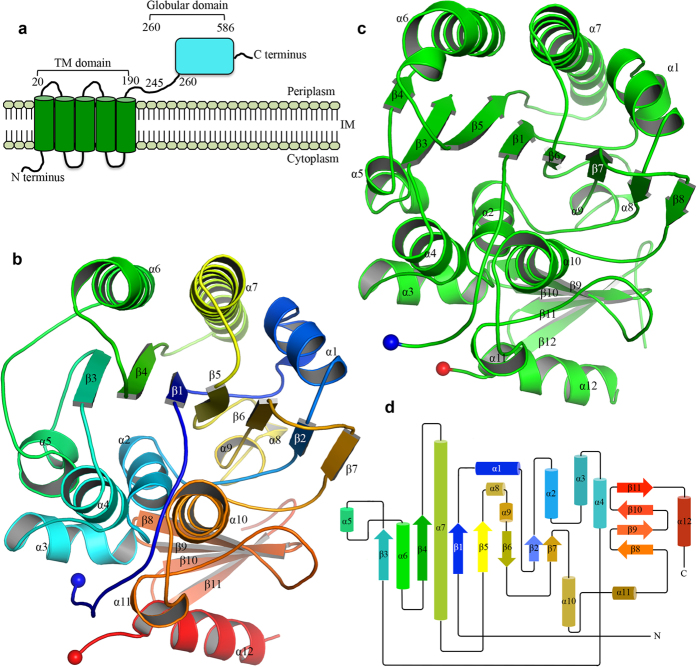
Two crystal structures of cardiolipin transport protein PbgA. The structures of PbgA resemble those of arylsulfatases and lipoteichoic acid synthases. (**a**) Scheme of PbgA of *S. typhimurium* strain LT2. (**b**) Cartoon representation of crystal structure of *St*PbgA245-586. The PbgA structure is colored in rainbow. (**c**) Cartoon representation of crystal structure of *E*CPbgA245-586 in green. (**d**) Scheme representation of structure of *St*PbgA245-586.

The PbgA (the original name YejM) protein was predicted to be an inner membrane protein containing five transmembrane domains (residues 1–190) and a C-terminal globular domain (residues 191–586) (Fig. 1a). To crystallize the PbgA proteins, the full length PbgA proteins and two N-terminal transmembrane domains truncation constructs (residues 191–586, and 245–586) named PbgA191-586 and PbgA245-586, from *S. typhimurium* Strain LT2 and *E. coli* K12 were cloned into pHISTEV plasmids. All the proteins were purified, and the PbgA245-586 from *S. typhimurium Str. LT2 (St*PbgA245-586) and *E. coli (Ec*PbgA245-586) were crystallized, while the purified PbgA191-586 showed degradation and crystallized with *in situ* limited proteolysis using elastase (Supplementary Fig. S2a,b). The crystal of *St*PbgA254-586 belongs to space group P2_1_ with cell dimensions a = 51.60 Å, b = 196.09 Å, c = 70.27 Å, α = γ = 90° and β = 95.70° (Table 1). The structure of *St*PbgA245-586 was determined to 1.64 Å resolution by single-wavelength anomalous dispersion (SAD) using selenomethionine (SeMet) incorporated protein. 28 selenium positions were identified in an asymmetric unit using SHELXD^33^. The electron density map was modified by SHELXE^34^,^35^. The initial model was built by Buccaneer^36^, and the full model was built manually using Coot^37^. Structure refinements were performed using REFMAC5 with the final model with the R_factor_ of 0.19 and R_free_ of 0.22 (Table 1).

There are four protomers of the *St*PbgA245-586 in the asymmetric unit. The structure of protomer A in the asymmetric unit is very similar to the other three protomers, B, C and D, with root-mean-square deviations (RMSD) of 0.285, 0.472 and 0.451 Å over 338 Cα atoms, respectively. The model of the *St*PbgA245-586 contains residues Q245-I563 and P568-A585, while the electron densities of the loop between I563-P568 are not visible, indicating that this loop is disordered (Fig. 1b,d).

The structure of *St*PbgA245-586 has the α/β fold, which looks like a burger, where helices α1, α6 and α7 are at the top, β-strands 1, 2, 3, 4, 5, 6, 7, 8, α8 and α9 form the second layer, α-helices 2, 4, 5 and 10 form the third layer, and β-strands 8, 9, 10, 11, α11 and α12 form the bottom layer (Fig. 1b).

The crystals of *St*PbgA191-586 belong to the space group P2_1_ with cell dimensions a = 41.49 Å, b = 196.39 Å, c = 45.66 Å, α = γ = 90° and β = 107.53°. The *St*PbgA191-586 structure was determined by molecular replacement to 2.19 Å resolution; residues 191–245 has no visible electron density so were either disordered or cleaved. There are four protomers in the asymmetric unit, where the protomer A is superimposed well with protomers B, C and D with RMSD of 0.2642 Å, 0.4338 Å and 0.5627 Å over 340 Cα atoms, respectively. The *St*PbgA191-586 structure is very similar to *St*PbgA245-586 structure with RMSD of 0.5945 over 340 Cα atoms (Supplementary Fig. S3a,b).

**Figure 2:**
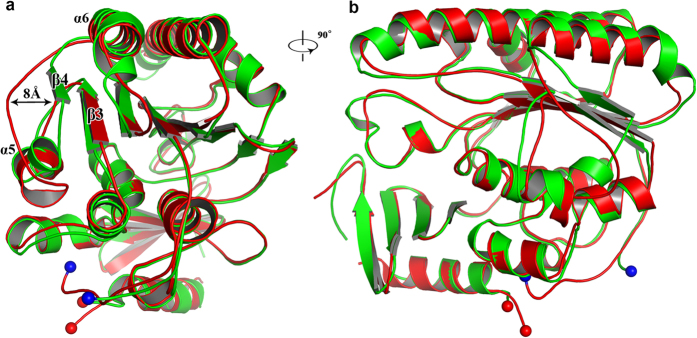
Comparison of structures of *St*PbgA and *Ec*PbgA. (**a**) *St*PbgA and *Ec*PbgA are superimposed well. *St*PbgA colored in red, and *Ec*PbgA colored in green. (**b**) Rotates 90° along y-axis relative to the left panel. Cα atoms of Loop S371-S360 moves away maximum of approximate 8 Å from of *St*PbgA to *Ec*PbgA, strongly suggesting that this loop may involve in CL binding.

The crystals of *Ec*PbgA245-586 belong to the space group of P3_1_2_1_ with cell dimensions a = b = 56.86 Å, c = 191.81 Å, α = β = 90° and γ = 120° (Table 1). The structure was determined by molecular replacement using the *St*PbgA245-586 structure as the search model to 1.67 Å resolution (Fig. 1c). There is one monomer in the asymmetric unit. The *Ec*PbgA-245-586 structure resembles *St*PbgA254-586 structure with a RMSD of 0.9264 Å over 340 Cα atoms. In particular, the β4 of *Ec*PbgA-245-586 moves away approximately 8 Å (Fig. 2a,b).

### PbgA resembles arylsulfatase family proteins and lipoteichoic acid synthase

**Figure 3:**
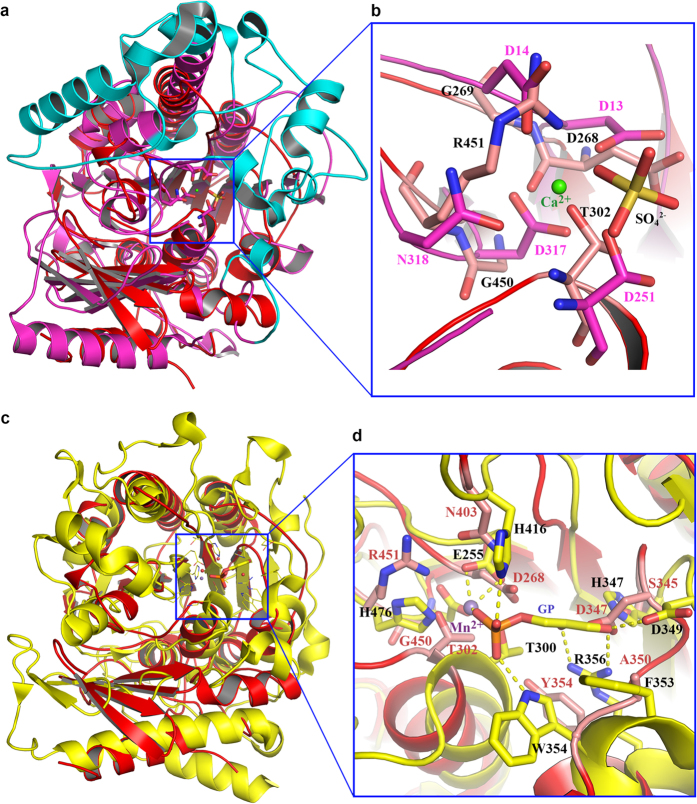
PbgA structure is similar to arylsulfatase and lipoteichoic acid synthase, but do not contain the enzyme catalytic residues. (**a**) *St*PbgA (red) is superimposed to arylsulfatase (matanga). The arylsulfatase has additional α-helices and loops (cyan), forming the substrate binding “gate”. (**b**) Superimposition of *St*PbgA and arylsulfatase at the active site. (**c**) StPbgA (red) is superimposed to lipoteichoic acid synthase (yellow). (**d**) Superimposition of StPbgA and lipoteichoic acid synthase at the active site.

To find similar structures to PbgA, we performed searches in the Protein Data Bank using the Dali server using the *St*PbgA245-586 structure as the template. The PbgA structure is very similar to sulfatase family enzymes, where arylsulfatase (PDB code 1HDH)^38^ has the Dali-score of 29.9 with root-mean-square deviation (RMSD) of 2.9 Å over 296 Cα atoms to the *St*PbgA245-586 structure (Fig. 3a). The sulfatases hydrolyse a variety of sulphate esters utilising an active site containing Cα-formylglycine, a conserved and oxidized cysteine or serine residue, and metal binding residues D317, N318, D13 and D14 (PDB code 1HDH) (Fig. 3b). Although the overall structure is similar by superimposition of the two structures, *St*PbgA245-586 does not have the cysteine or serine at the position of the Cα-formyglycine of the sulfatase. We also did not observe any cysteine or serine residue of *St*PbgA245-586 around the position of the sulfatase’s active site. Instead, the *St*PbgA245-586 structure has residues T302, G450, R451, G269 and D268 at the corresponding positions in the sulfatase active site, respectively (Fig. 3a,b); this is consistent with the report that the PbgA does not have the sulfatase activity^24^. The major differences between the two structures are that the sulfatase has additional α-helices^38^ and loops that form a “gate” to close the active site (Fig. 3a).

The structure of lipoteichoic acid synthase of *Staphylococcus aureus* (LtaS, PDB code 2W5T)^39^, is also very similar to the *St*PbgA245-586 structure, with the Dali score of 28.4 and a RMSD of 3.0 Å over 284 Cα atoms. Despite only 17% sequence identity to *St*PbgA, LtaS is also a membrane protein with five transmembrane domains and a globular soluble domain with a structure strikingly similar to the *St*PbgA245-586 structure (Fig. 3c). The LtaS structure has revealed the active site pocket for binding a Mn^2+^ ion and Beta-Glycerol pho-sphate (sn-glycerol-2-phosphate), where residues T300, H476 and E255 bind Mn^2+^ ion, and H416, W354, F353, D349, H347 and R356 are involved in the Beta-Glycerol pho-sphate (sn-glycerol-2-phosphate) binding (Fig. 3c,d). As described above, the corresponding residues T302, G450, R451 and D268 of *St*PbgA are unlikely to form the metal binding site (Mn2^+^). The residues H347, D349, R356 and W354 of LtaS involving in Beta-Glycerol pho-sphate (sn-glycerol-2-phosphate) binding were confirmed to be important for the activity of LtaS^39^. The arrangement of the corresponding residues N403, S345, D347, A350 and Y354 of *St*PbgA does not correspond to the Beta-Glycerol pho-sphate (sn-glycerol-2-phosphate) binding site (Fig. 3d).

### *St*PbgA245-586 does not bind metal ions and Beta-Glycerol pho-sphate (sn-glycerol-2-phosphate)

To confirm whether *St*PbgA245-586 binds heavy atoms and Beta-Glycerol pho-sphate (sn-glycerol-2-phosphate), we have performed co-crystallization the *St*PbgA245-586 in presence of the 20mM Mn^2+^ and 20 mg/ml sn-glycerol-2-phosphate. However, no extra electron density is visible in the solved structure, suggesting that PbgA may not bind metal or sn-glycerol-2-phosphate.

### Potential CL binding site

**Figure 4:**
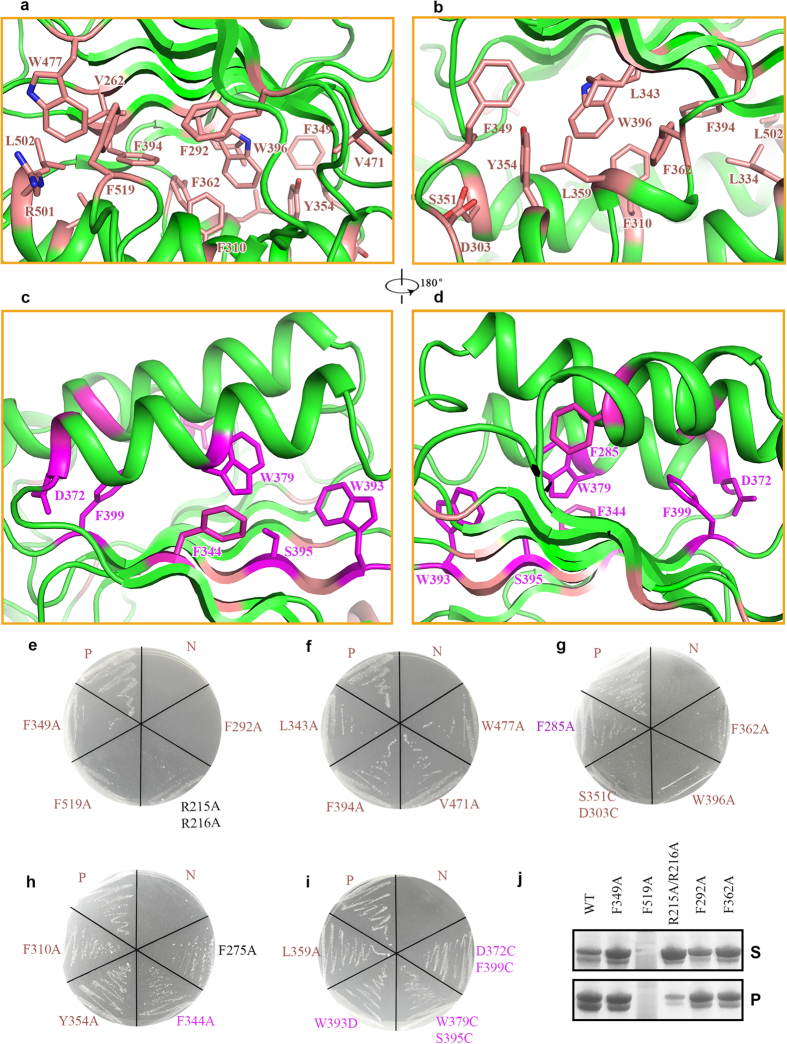
PbgA potential CL binding site. (**a**) the CL potential binding site between layers 2 and 3. The hydrophobic residues are colored in deep salmon. (**b**) Rotation 180° along y axis relative to the left panel. (**c**) Hydrophobic residues between layer 1 and 2 are colored in purple, which may do not involve in CL binding. (**d**) Rotation 180° along y axis relative to the left panel. (**e–i**) The functional assays of PbgA mutants and the drug resistance against 50 ug/ml vancomycin. (**j**) The CL binding assays were performed by co-sedimentation. All functional assays performed at least three times.

From the structures of the sulfatase and LtaS, their active sites are between the second layer and the third layer. In PbgA, the second layer consists of the β-sheet formed by strands 1–7 and the third layer is composed of of α-helices 2, 4, 5 and 10. This region is a highly hydrophobic core, which is formed by the hydrophobic residues L343, W396, F394, V262, W477, L473, V471, and F292 from the β-sheet of the second layer, and residues F349, Y354, L334, F310, L309, and F519 from the α-helices and loops of the third layer (Fig. 4a,b). PbgA transports CL from the IM to the OM^24^. We speculate that the hydrophobic core between the second and third layers might be the CL binding site. To explore this possibility, single alanine substitutions of residues L359, F292, F349, F519, L334, L343, F394, W396, V471 and W477, as well as a double alanine substitution R215/R216, were generated, and the PbgA global domain (191–586) inactivated strain NRD183 was used for the functional assay^40^.

The deletion of PbgA periplasmic globular domain causes the outer membrane defect, which increases the OM permeability. The NRD183 strain was unable to grow in 50 μg/ml vancomycin, and the full length PbgA can rescue the NRD183 strain. The F292A mutant is lethal, and F349A, R215A/R216A, W396A, F362A and F275A severely impaired bacterial growth in 50 μg/ml vancomycin (Fig. 4e,g,h), while mutants L343A, F394A, V471A, W477A have minor effect on cell growth (Fig. 4f), and F310A, Y354A, L359A has no effect on cell growth (Fig. 4i), suggesting that residues F292, F349, R215/R216, W396, F362 and F275 are important for CL transport. The functional assay result of R215A/R216A mutant is consistent with the previous report that the residues R215/R216^24^ are involved in CL binding (Fig. 4e).

Residues F344, W379, W393 and F399 are located in the hydrophobic core between the α-helices 6 and 7 of the first layer and the β-strands 1 and 4 of the second layer of *Ec*PbgA (Fig. 4c,d). In contrast, single mutant F344A, W393D, and double mutants F399C/D372C and W397C/S395C did not have any impact on cell growth, strongly suggesting that these residues are not involved in the CL binding and transfer (Fig. 4h,i).

CL binding residues were further confirmed using CL co-sedimentation assays. Mutant proteins (F349A, R215A/R216A, F292A and F362A) of *Ec*PbgA 191–586 were over-expressed and purified. Mutants F349A, R215A/R216A, F292A, F362A have reduced CL co-sedimentation compared to the wild type (Fig. 4J); this is consistent with our functional assays, supporting that CL binding by these mutants is impaired. The F519A mutant forms an inclusion body so could not be assessed by CL co-sedimentation.

### Potential CL-binding switch lid

**Figure 5:**
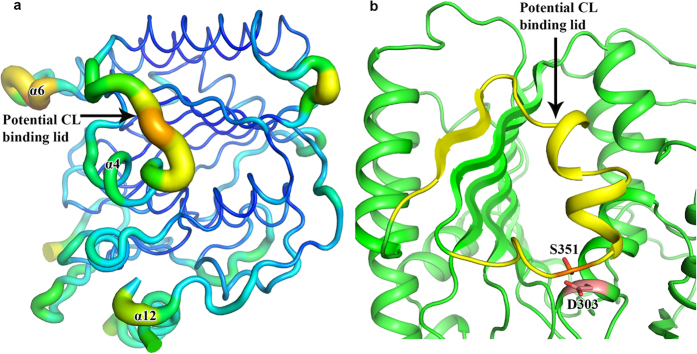
Potential CL binding lid. (**a**) The PbgA loop consisting of residues D347-Q370 has higher B-factor comparing to the local residues, suggesting that the loop is flexible and may be a lid for the CL binding. (**b**) Cartoon representation of potential CL binding lid colored in yellow and the double cysteine mutation residues S351C/D303C may form a disulphide bond and prevent the lid to open for CL binding.

Comparison of the crystal structures of *Ec*PbgA245-586 and *St*PbgA245-586 revealed a conformational change of the loop between helices α5 and α6 (Fig. 2a). In *Ec*PbgA245-586 this loop closes the hydrophobic core between the second (β-strands 1–8, α8 and α9) and third layers (α-helices 2, 4, 5 and 10) and the residues P365-R368 form β-strand 4 to interact with β-strand 3, whereas the strand β4 is disordered and away from the hydrophobic core between the second and third layers in *St*PbgA245-586 (Figs 1b,c and 2a). Residue F349 is solvent exposed and F362 is located in the hydrophobic core in *Ec*PbgA245-586, while the residue F349 rotates toward the hydrophobic core and the F362 is solvent exposed in *St*PbgA245-586 (Fig. 4a,b). Analysis of the *St*PbgA245-586 structure revealed that there is a loop formed by residues D347-Q370 that has high B-factors between helices α4 and α6, indicating that this loop is flexible and may act as a lid for the CL binding and transport (Fig. 5a). To explore this possibility, we generated a double cysteine mutant S251C/D303C, which would lock the switch and significantly influence the CL binding. Indeed the mutant S251C/D303C impairs the cell growth (Fig. 4g).

## Discussion

The OM of Gram-negative bacteria is an asymmetric membrane composed of phospholipids and lipopolysaccharide. The lipids in the OM play important roles in the pathogenesis and drug resistance of Gram-negative bacteria. Despite the extensively studied LPS transport pathway in Gram-negative bacteria, the mechanisms for phospholipids transport and assembly in the OM remain to be characterized at the structural level.

**Figure 6:**
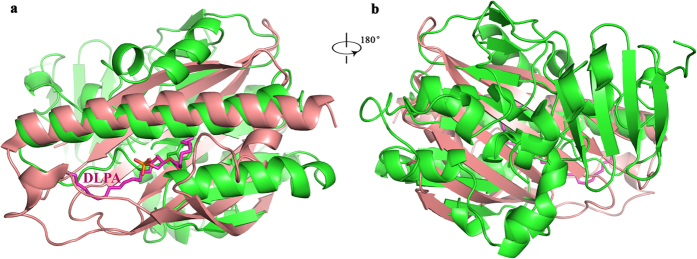
The PbgA structure is quite different from the UPS1 of mitochondria of yeast. (**a**) Only the long helix α7 of PbgA is superimposed well with the helix α3 of UPS1. (**b**) Rotation 180° along y axis relative to the left panel. DLPA, 1,2-dilauroyl-sn-glycero-3-phosphate (12:0–12:0).

CL plays an essential role in OM remodelling in bacteria and is critical for mitochondrial function^41^. PbgA transports CL from the IM to the OM in Gram-negative bacteria^24^. Mitochondria evolved from bacteria and the UPS1/Mdm35 complex has been identified for phospholipid transfer from the OM to the IM in yeast. The recent structures of the UPS1 of yeast in complex with phospholipids have been determined^29^,^30^. The structure is quite different from those of PbgA with a RMSD of 3.7 Å over 62 Cα atoms (Fig. 6a,b). The UPS1 contains a concaved 7-antiparellel stranded β-sheet, which wraps PA molecules inside the cavity. Only the long helix α7 of PbgA is superimposed well with the helix α3 of UPS1; the conformation of the β-sheet at the second layer of PbgA is different from that of the UPS1, which does not fold to form a cavity (Fig. 6a), indicating that this area between the first and second layers of PbgA may not be the CL binding site. Indeed, the functional assays using alanine substitution of aromatic residues between the first and the second layer showed that all the mutants did not impair bacterial growth, which suggest that the PbgA-mediated transport of CL is different from UPS1-mediated transport of PA in mitochondria.

The structure of *Ec*PbgA245-586/*St*PbgA245-586 is strikingly similar to arylsulfatases and lipoteichoic acid synthases, but it lacks their enzymatic activities^39^. Metal ions, such as Mg^2+^, Mn^2+^ or Ca^2+^ are required for their catalytic function. Superimposition of PbgA structures over those of arylsulfatase and lipoteichoic acid synthase suggested that PbgA is unlikely to form the required metal ion-binding site found at the active sites of arylsulfatase and lipoteichoic acid synthase.

The functional studies showed that single hydrophobic residue mutants F292A, F349A and F362A caused the cell death (Fig. 4e) or impaired cell growth in 50 μg/ml vancomycin, indicating that these residues are involved in CL transport. This was further confirmed by the co-sedimentation assays (Fig. 4J). All these residues are located in the hydrophobic core between the layers 2 and 3 of PbgA245-586, strongly suggesting that this hydrophobic core is the potential CL binding site. Additionally, single mutant F344A, W393D, and double mutants F399C/D372C and W397C/S395C between the first layer and the second layer did not have any influence on cell growth (Fig. 4h,i), which further confirms that the hydrophobic space between layer 2 and 3 may be responsible for the CL transport.

There is a significant conformational change of residues D347-Q370 between the *Ec*PbgA245-586 and *St*PbgA245-586 (Fig. 2a), and these residues’ B-factors are higher than neighbouring residues (Fig. 5a), indicating that they are flexible. We speculated that this region may be the lid for the CL binding, which can open to allow the CL access the binding site between the second and third layers. The double cysteine mutant S351C/D303C may form a disulphide bond and prevent the lid to open for CL binding (Fig. 5b). This mutant is lethal, suggesting that the loop may be the lid for the CL binding (Fig. 4g). The B-factors of the last α-helix 12 are also higher compared to other local residues (Fig. 5a), indicating that this helix is flexible, which may suggest that it interacts with other proteins or OM components.

Phospholipid transport and assembly in the OM is a basic biological process. Our structural and functional studies have revealed that the hydrophobic core between the layer 2 and 3 may be the CL binding site and that the residues D347-Q370 may form a lid for the CL transport in the structures of PbgA245-586 of *E. coli* and *S. typhinium*, and. This is a first structure of a bacterial protein involved in phospholipid transport from the inner membrane to the OM. This work is important for understanding how bacteria pathogenesis and drug resistance is achieved by remodelling the OM, in addition to clarifying how Gram-negative bacteria assemble their OM.

## Experimental Procedures

### Cloning, expression, and purification of PbgA_245–585_

The pbgA gene (yejM) encoding PbgA245-586 and PbgA191-586 from *S. typhimurium* strain LT2 and *E. coli* strain K-12 were amplified and inserted into plasmid pEHISTEV with a NcoI restriction sites at 5′-end and EcoR1 sites at 3′-end respectively. This plasmid includes a hexahistidine tag (6× His) and a tobacco etch virus (TEV) protease cleavage site between the Histag and the N-terminus of the cloned genes. The recombinant plasmids were transformed into soluble BL21(DE3) strain (Novagen) for protein expressions. The transformed soluble BL21(DE3) cells were grown in Luria broth (LB) supplemented with antibiotic (Kanamycin 50 μg/ml) at 37 °C until the optical density of the culture reached 0.5–0.8 at a wavelength of 600 nm (OD_600nm_). The proteins were induced by addition of 0.1 mM isopropyl β-d-thiogalactopyranoside (IPTG) and incubated for 12 hours at 20 °C. For over-expression of selenomethionine (SeMet) labeled PbgA245-586, the protein was expressed in M9 medium supplemented with SeMet (Generon) to a final concentration of 100 μg/ml using the methionine inhibition method^42^. The cells were harvested by centrifugation at 5000 × g for 20 mins, resuspended in buffer containing 20 mM Tris-Cl, pH 7.8, 10% glycerol and 500 mM NaCl, supplemented with cOmplete (Roche), 1 mM DNase (Sigma-Aldrich) and 1 mM phenylmethylsulphonyl fluoride (PMSF, Sigma-Aldrich). The cells were broken using a cell disruption at 30,000 psi (Constant Systems Ltd). The Cell debris was removed by centrifugation at 120,000 × g for 25 mins at 4 °C. The supernatant was then being loaded onto a nickel-nitrilotriacetate affinity resin column (Ni-NTA, Qiagen) and the column was washed with 20 mM Tris-Cl, pH 7.8, 500 mM NaCl, 30 mM imidazole and 10% glycerol. The recombinant proteins were eluted with 20 mM Tris-Cl, pH 7.8, 500 mM NaCl, 300 mM imidazole and 10% glycerol. The protein buffer was changed to 20 mM Tris-Cl, pH 7.8, 500 mM NaCl, 10% glycerol and 10 mM imidazole using a desalting column (Hi-Prep^TM^ 26/10, GE Healthcare) to prevent protein precipitation. The (6 × His) tag was removed by TEV protease, and the PbgA protein was obtained by applying the samples through a Ni-NTA column. The protein was further purified using size exclusion chromatography with a HiLoad 16/60 Superdex 200 prep grade column (GE Healthcare) in a running buffer containing 20 mM Tris-Cl, pH 7.8 and 150 mM NaCl. Fractions with the highest purity of PbgA were pooled and concentrated to 10 mg/ml.

### Crystallization and Data collection

Protein crystallization trails were performed using 1 μl of protein mixed with 1 μl of reservoir solution by the sitting-drop vapour diffusion technique at room temperature. The best crystals of SeMet incorporated *St*PbgA245-586 were obtained in 0.1 M Bis-tris pH 5.5 and 16% PEG3350. The best crystals of in *Ec*PbgA245-586 were obtained in 0.2 M lithium sulphate, 0.1 M sodium acetate pH 4.3 and 48% PEG400. The best native crystals of *St*PbgA191-586 were obtained in 0.1 M Bis-tris pH 6.5 and 25% PEG3350. The crystals were harvested after 21 days and cryoprotected by supplementing the crystallization solution with 20% glycerol in the crystallization solutions, before being flash frozen in liquid nitrogen. Single-wavelength anomalous dispersion (SAD) data were collected at Diamond Light Source, UK at beam station I04. All data were processed using XDS^43^ and the *St*PbgA245-586 crystals that diffracted to 1.69 Angostram resolution belong to space group P2_1_ with unit-cell dimensions: a = 52.6 Å, b = 196.09 Å, c = 70.27 Å and β = 95.79°. The native *Ec*PbgA245-586 crystal data were collected to 1.65 Å resolution and the crystals belong to space group *P*3_1_2_1_ with unit-cell dimensions: a = 56.86 Å, b = 56.86 Å, c = 191.81 Å and γ = 120°.

### Structure determination

The structure of *St*PbgA245-586 was solved by the SAD method using SHELX suite^34^. The initial model of the *St*PbgA245-586 was built automatically using the program Buccaneer^36^ with the phases obtained form the SHELX^34^. The native structure (*Escherichia coli* strain K-12) was determined by molecular replacement using structure from *Salmonella* as a search model using Phase^44^. The structures were manually built by using Coot^37^ and the structures were refined using REFMAC5^45^. The structures were validated by Molprobity^46^. The statistics of the data collection and the structure refinement are summarized in Table 1.

### Site-directed mutagenesis and functional assays

All single or double mutations were generated following the protocol^47^. The mutations were amplified by PCR using Q5^®^ hot start high fidelity DNA polymerase (New England) and the pACYCDuet plasmid (Novagen), containing a C-terminal (6× His) tag of PbgA *E. coli* gene with an NcoI restriction site at the 5′-end and Hind III site at the 3′-end as the template for the *pbgA* mutagenesis.

The NRD183 strain still contains the transmembrane domain and only the periplasmic global domain is depleted. The NRD183 also contains a plasmid pNRD217, which carries the whole pbgA gene under pBAD control. As the NRD183 contains the N-terminal transmembrane domain, the strain is a conditional lethal strain. The strain is killed in presence of 50 ug/ml vancomycin without PbgA expression.

The mutations were confirmed by DNA sequencing. Once the full length of the PbgA was shown to rescue the NRD183 strain, these single or double mutants were transformed into *E. coli* NRD183^40^, respectively. The transformed *E. coli* cells were grown on LB agar plate supplemented with antibiotics (kanamycin 50 μg/ml, chloramphenicol 34 μg/ml, and ampicillin 50 μg/ml). Single colonies of each transformation was inoculated into 5 ml LB medium supplemented with above antibiotics and 0.2% L-arabinose. Subculture cells were used for functional assay. *E. coli* NRD183 with the empty plasmid pACYCDuet was used as a negative control. For functional assays, cells were harvested, washed one time and diluted in sterile LB medium to an absorbance (OD600 nm) of 0.5 and streaked onto LB agar plates supplemented with vancomycin 50 μg/ml, kanamycin 50 μg/ml and chloramphenicol 34 μg/ml and 5mM IPTG. Cell growths were recorded after overnight culture in 37 °C. All the assays have been repeated in triplicate.

### Protein-Lipid co-sedimentation assays

Cardiolipins (CL) in chloroform (Sigma) was dried under nitrogen gas, and then re-suspended in 20 mM Tris (pH 7.8), and 150 mM NaCl. Subsequently, the CL was sonicated until the solution to be clear. The mixture (Lipid-protein) were incubated at 37 °C for 1 hour at a 10:1 molar ratio (20 mM: 2 mM), respectively following the reported method^24^. The reactions were centrifuged at 13.2 k rpm for 10 min. The supernatants were collected and the pellets were re-suspended in 20 μl of buffer in 20 mM Tris (pH 7.8), and 150 mM NaCl. The pellets (10 μl) and supernatants (10 μl) were boiled in the SDS-containing sample buffer and separated on 12% SDS Tris-HCl PAGE. The proteins were visualized by Coomassie staining.

## Additional Information

**Accession codes:** The atomic coordinates and structure factors of *Ec*PbgA245-585, *St*PbgA245-585 and *St*PbgA191-585 are deposited at the Protein Data Bank under access code 5I5H, 5I5D and 5I5F.

**How to cite this article**: Dong, H. *et al*. Structural insights into cardiolipin transfer from the Inner membrane to the outer membrane by PbgA in Gram-negative bacteria. *Sci. Rep.* **6**, 30815; doi: 10.1038/srep30815 (2016).

## Supplementary Material

Supplementary Information

## Figures and Tables

**Table 1:** Data collection and structure refinement statistics.

Data collection	*Ec*PbgA245	*St*PbgA191	*St*PbgA245 (SAD)
**Resolution (Å)**	47.83–1.67 (1.71 −1.67)	65.22–2.19 (2.25–2.19)	65.85–1.64 (1.68 −1.64)
**Wavelength (Å)**	0.9795	0.9795	0.9795
**Space Group**	P3_1_2_1_	P2_1_	P2_1_
**Completeness (%)**	99.6 (99.2)*	99.4 (99.4)	95.1 (89.2)
**I/σ**	17.8 (1.1)	5.8 (1.8)	15.5 (1.1)
**Multiplicity**	9.6 (7.6–9.9)	6.1 (6.4–4.8)	11.8 (13.1–6.1)
**CC1/2 (%)**	100 (59.5)	98.7 (54.5)	99.9 (49.5)
**Unique reflections**	44003 (4034)	51747 (5044)	66831 (6540)
**R**_**merge**_ **(%)**	14.3 (26.40)	16.50 (27.80)	15.2 (25.03)
**Unit Cell (Å)**	a = b = 57.42, c = 191.31	a = 51.48 b = 195.65, c = 70.92	a = 51.60 b = 196.09, c = 70.27
	α = β = 90°, γ = 120°	α = γ = 90°, β = 96.39°	α = γ = 90°, β = 95.79°
**Refinement**			
&**R**_**factor**_	0.18 (0.21)	0.20 (0.23)	0.19 (0.20)
**R**_**free**_	0.21 (0.25)	0.24 (0.27)	0.22 (0.24)
**Number of atoms**			
Protein	2604	10724	10745
Waters	498	961	1243
**R.M.S. deviation**			
Bond lengths (Å)	0.014	0.02	0.017
Bond angles (°)	1.90	1.93	1.80
**Ramachandran statistics**			
Favoured (%)	97	93	95
Allowed (%)	3	7	5
**PDB code**	5I5H	5I5F	5I5D

^*^Values in parentheses are represents for the highest-resolution shell.

^&^**R**_factor_ = Σ || **F**_**obs**_**|−| F**_**cal**_**||**/Σ |F_obs_|, where F_obs_ and F_cal_ are observed all reflection measured and calculated currently model as structure factors, respectively. ^R^free is calculated using 5% of total reflections, which is randomly selected not used in refinement.
