# Supplementary material for: Structural insights into cardiolipin transfer from the Inner membrane to the outer membrane by PbgA in Gram-negative bacteria
Source: Sci Rep. 2016 Aug 4;6:30815. doi: 10.1038/srep30815 (PMC4973235; doi:10.1038/srep30815)
Supplement: Supplementary Information [file srep30815-s1.pdf]

Structural insights into cardiolipin transfer from the Inner membrane to the outer membrane by PbgA in Gram-negative bacteria

Haohao Dong[1§], Zhengyu Zhang[1§], Xiaodi Tang[2§], Shihai Huang[1,3], Huanyu Li[1], Bo Peng[1], Changjiang Dong[1*]

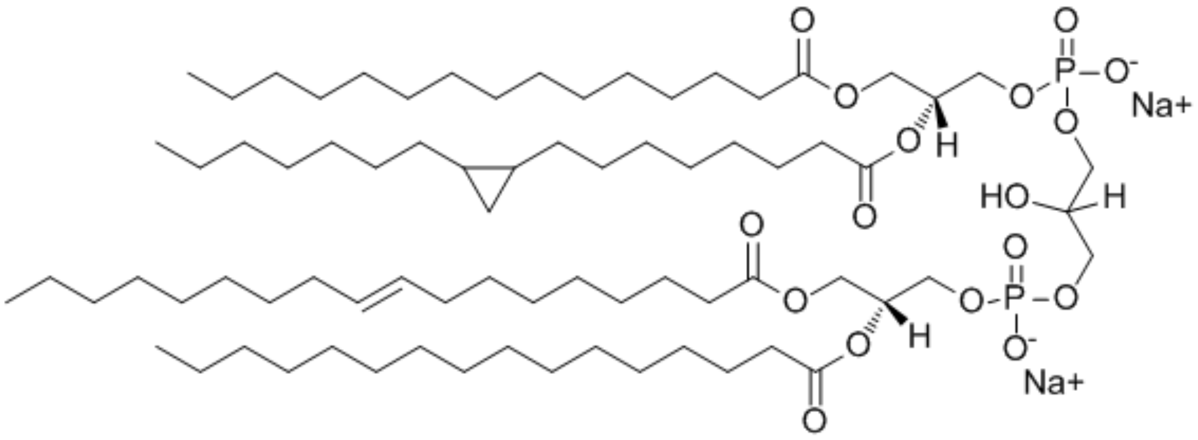

Figure S1. Structure of cardiolipin.

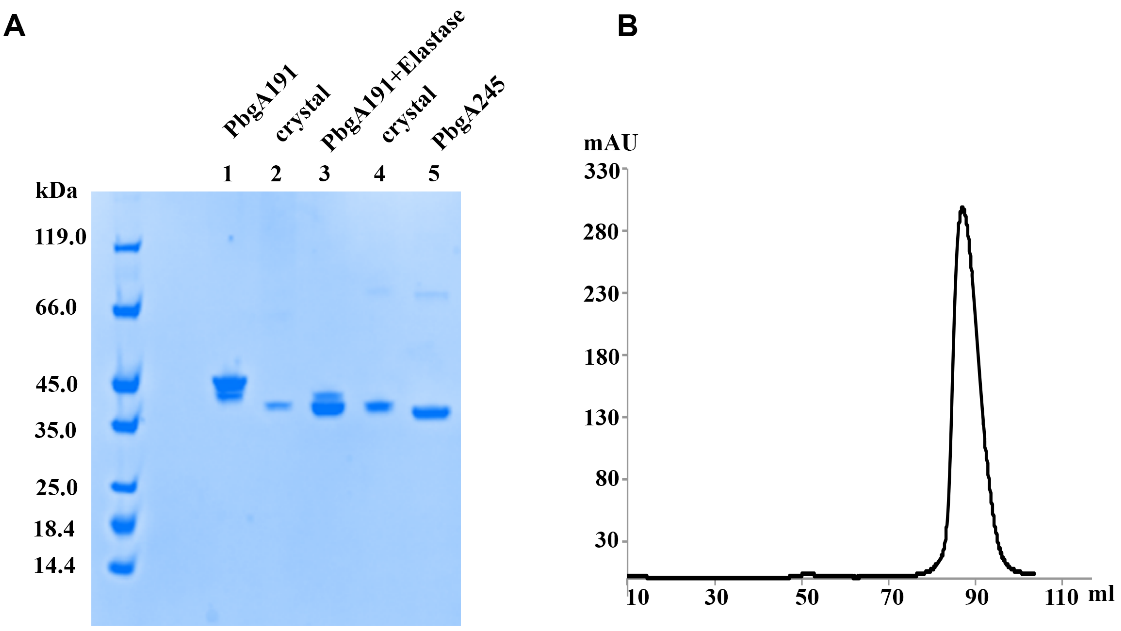

Figure S2. SDS-PAGE of purified *St*PbgA and crystals and the gel filtration chromatography of the *St*PbgA245-586. (A) SDS-PAGE of purified PbgA proteins and crystals from the *Salmonella*. 1, purified *St*PbgA191-585 protein, 2, crystals from the *St*PbgA191-585 protein, indicating the protein was cleaved during crystallization. 3, *St*PbgA191-585 was digested with elastase for 2hrs at room temperature at 1:100 mole ratio.4, crystals from the *St*PbgA191 after elastase digestion. 5. Purified protein *St*PbgA245-585. (B) The gel filtration chromatograph of the *St*PbgA245-586.

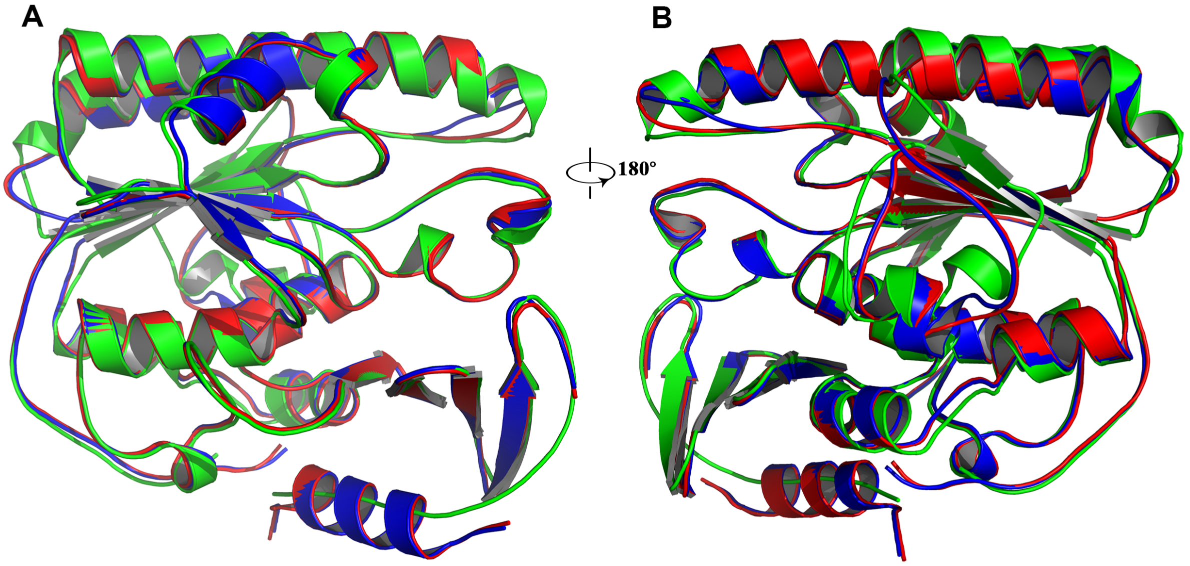

Figure S3. The structures of *St*PbgA245-586, *St*PbgA191-586 and *Ec*PbgA245-586 are very similar. The three structures of *St*PbgA245-586 (red), *St*PbgA191-586 (blue) and *EC*PbgA245-586 (green) are superimposed well with RMSD of 0.405 Angstrom over 338 Ca atoms between *St*PbgA245-586, *St*PbgA191-586, and RMSD of 1.08 angstrom over 310 Ca atoms between *St*PbgA245-586 and *Ec*PbgA245-586. (A) The three structures are superimposed. (B) The figure a rotates 180° along y axis.
